# The Role of Ischemia-modified Albumin as a Biomarker in Preeclampsia

**DOI:** 10.1055/s-0040-1709662

**Published:** 2020-03

**Authors:** Süleyman Serkan Karaşin, Tayfur Çift

**Affiliations:** 1 Department of Obstetrics and Gynecology, Health Sciences University Bursa Yuksek Ihtisas, Education and Research Hospital, Bursa, Turkey

**Keywords:** preeclampsia, ischemia modified albumin, hypertension, pregnancy

## Abstract

**Objective** Ischemia-modified albumin (IMA)is a modified type of albumin protein that is formed under oxidative stress. This study aims to compare the levels of serum IMA between normotensive and preeclamptic pregnancies and to evaluate the relationship between the severity of the disease.

**Methods** A total of 90 pregnant women aged between 18 and 45 years participated in this cross-sectional study. The levels of serum IMA were measured by enzyme-linked immunosorbent assay in 30 preeclamptic pregnant women with the severe signs of the disease, 30 preeclamptic pregnant women, and 30 normotensive pregnant women.. The study was designed as a cross-sectional clinical study.

**Results** When the demographic characteristics were examined, statistically significant differences were found between the groups in terms of age, gestational week at birth and blood pressure. Age was higher in the preeclampsia with signs of severity group than in the normotensive group (*p* = 0.033). Pregnancy week was significantly the lowest in the preeclampsia with the severity signs group (*p* = 0.004). In normotensive patients, IMA levels were lower than in the preeclampsia groups (*p* < 0.001) but there was no significant difference in terms of severity of disease (*p* = 0.191). According to laboratory data; only the creatinine level was significantly different between the groups.

**Conclusion** The levels of serum IMA were higher in patients with preeclampsia than in healthy pregnancies. However, there was no significant correlation in terms of preeclampsia severity; more extensive, prospective and long-term studies are needed.

## Introduction

Complicating 2 to 8% of pregnancies, pre-eclampsia, along with the other hypertensive disorders of pregnancy, is a major contributor to maternal mortality worldwide. Although maternal mortality is much lower in high-income countries than in developing countries, 16% of maternal deaths can be assigned to hypertensive disorders.^1^

Preeclampsia is a pregnancy-specific hypertensive disease with multisystem involvement. It usually occurs after 20 weeks of gestation, most often near term, and can be superimposed on another hypertensive disorder.^2^

Although the pathogenesis of preeclampsia is not clear, the failure of the trophoblastic invasion of spiral arteries plays a role in the pathogenesis. In this respect, ischemia-modified albumin (IMA), which is one of the serum biomarkers of tissue damage and myocardial ischemia, maybe a potential marker.^3^

**Fig. 1:**
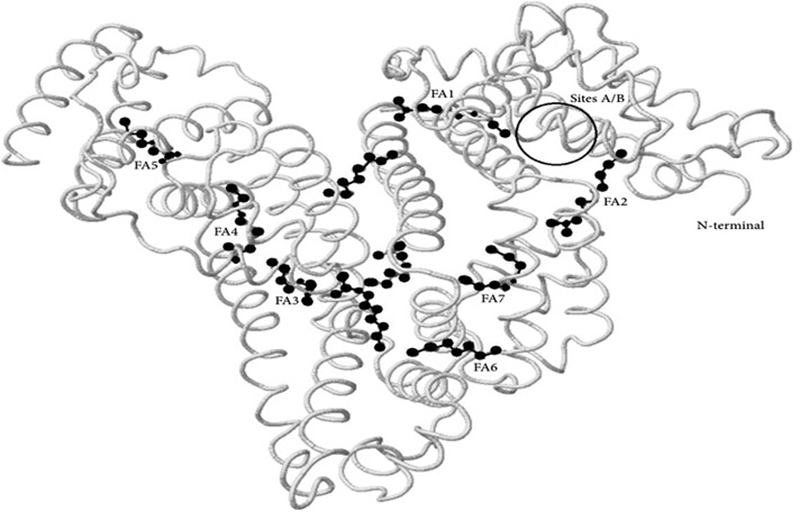
Location of the seven major fatty acid (FA)-binding sites of human serum albumin, with the main cobalt binding sites. **Source:** Anwaruddin et al.^4^

Recently, a modification of ischemia-dependent human serum albumin (HSA) has been proposed as a serum biological marker of myocardial ischemia. Under physiological conditions, the amino-terminal end of the HSA (N-terminal) binds metals such as cobalt, copper, and nickel. During ischemia, several changes occur at the amino-terminal end (N-terminus) of the HSA; such changes are probably caused by oxidative free radicals, which reduce the binding properties of the transition metals, especially cobalt. This new and chemically-modified molecule is called IMA (Fig. 1).^4^

Serum IMA appears to be elevated in normal pregnancy. Guven et al^5^ suggested that this may be due to physiological oxidative stress in pregnancy. Normal pregnancy is characterized by a significant maternal adaptive response, including inflammation activation, endothelial cell activity, and activation in maternal coagulation as well as the production of many pro-oxidants and vasoactive substances by plasma.^6^

The early stages of pregnancy are associated with oxidative stress between the maternal decidua and placental villi. This state of hypoxia exists before the development of maternofetal circulation. Low oxygen pressure at this stage stimulates normal cell differentiation and proliferation of cytotrophoblasts. This ensures placental placement before the rapid fetal growth period in the second half of pregnancy.^7^

Normal pregnancy is always under stress to meet the growing demands of the developing fetus. Various metabolic changes occur with vascular remodeling in the maternal system. Therefore, pregnancy is always associated with oxidative stress and reactive oxygen radicals (ROS). Ischemia-modified albumin produced by ROS is found as a sensitive and early biochemical marker of ischemic heart disease and is now used as an important marker to differentiate ischemic and non-ischemic pathologies. Hypoxic-ischemic pregnancy may cause an increase in serum IMA.^8^

For these reasons, it seems worthwhile to test the IMA molecule in cases of preeclampsia, including hypoxia and ischemia in its pathogenesis.

As of today, IMA has been rarely described and has never been established in the context of the severity of preeclampsia. Our study aimed to evaluate IMA in preeclamptic pregnant, normotensive pregnant and terms of the severity of the disease.

## Methods

The present study was conducted at the Department of Obstetrics and Gynecology from the University of Health Sciences Bursa Yuksek Ihtisas Education and Research Hospital, Yildirim, Bursa, Turkey, in a 5-months period between August 2017 and December 2017, and it is designed as a cross-sectional clinical study. The participants were randomly selected and divided into 3 groups among pregnant women over the age of 18, who had no additional disease, regardless of the gestational week, whose labor had begun and were admitted to the delivery room. We included 90 participants in our study. We had three groups in our study. The first group consisted of 30 preeclamptic pregnant women; the second group consisted of 30 preeclamptic pregnant women who had the severe features of the disease; and the control group consisted of 30 normotensive pregnant women. The participants were followed up in the delivery room. We collected serum samples participants who were decided to give birth just before the labor started. The study was approved by the ethics committee of clinical research of the Uludag University Faculty of Medicine, Bursa, Turkey. For accurate measurement of blood pressure, we followed the European Society of Hypertension/ European Society of Cardiology (ESH/ESC) guidelines for the management of arterial hypertension 2013. Brachial artery blood pressure was evaluated. The blood pressure measurement of each patient at 5 minutes of rest while sitting, with the cuff at heart level brachial artery blood pressure was evaluated. If there was a difference of more than 5 mm Hg between the repetitions of at least two systolic or diastolic blood pressures, it was appropriate to repeat the process with two additional measurements. Each measurement was made with a proper rest break. The diagnosis of preeclampsia was based on the standard criteria in the guidelines published by the American College of Obstetricians and Gynecologists (ACOG) in 2013. According to this:

- ≥ 140 mm Hg systolic or ≥ 90 mm Hg diastolic on 2 occasions at least 4 hours apart, after 20 weeks of gestation, in a woman with a previously normal blood pressure/or measurement of systolic blood pressure ≥160mm Hg after 20 weeks of gestation, or measurement of diastolic blood pressure ≥ 110 mm Hg. In addition, if proteinuria is ≥ 0.3 g or protein/creatinine ratio is ≥ 0.3 mg/mg in 24-hour urine or if quantitative measurement cannot be performed with dipstick ≥1 + protein detection. Or;
- New-onset hypertension and the onset of any of the following (proteinuria or absent)
  (1) Platelet count < 100,000 / micro L
  (2) Serum creatinine >1.1 mg/dL (97.2 micromole/L) or doubling creatinine concentration without any other renal pathology
  (3) At least twice the upper limit of normal transaminase levels
  (4) Pulmonary edema
  (5) Cerebral or visual symptoms.^2^

The following criteria were used in 2013 by the American College of Obstetricians and Gynecologists (ACOG) for the diagnosis of severe features of preeclampsia:

- Systolic blood pressure ≥ 160 mm Hg and/or diastolic blood pressure ≥ 110 mm Hg at two separate measurements at least 4 hours apart when the patient is at bed rest or;
- Platelet count < 100,000 / micro L or;
- Progressive renal failure (serum creatinine > 1.1 mg / dL or doubling creatinine concentration without other renal pathology) or;
- Impaired liver function, at least twice the upper limit of normal transaminase levels, widespread right upper quadrant pain, epigastric pain or;
- Pulmonary edema or;
- Cerebral or visual symptoms^2^; the presence of any of the symptoms describe the severe features of the disease.

Pregnant women with smoking, chronic hypertension, autoimmune disease, diabetes, hypercholesterolemia and those younger than 18 years were excluded from the study. No known disease was noted that would affect the platelet count and liver enzyme levels. The control group was formed from healthy pregnant women according to the gestational weeks and demographic characteristics of the patient group. Obstetrical evaluations of all pregnant women were performed and fetal development was evaluated according to ultrasonographic measurements. Also, we used the enzyme-linked immunosorbent assay (ELISA) method to evaluate IMA, which is a small number in the literature.

## Sampling the Materials

Maternal blood samples were obtained from all preeclamptic patients who were admitted to our clinic. Blood samples were taken from healthy pregnant women before the onset of any treatment. In the sitting position, 3cc blood samples were taken to anticoagulant tubes after sterilization of the forearm antecubital region. All the pregnant women included in the study had not started labor. Samples were centrifuged at 5,000 RPM for 5 minutes. After centrifugation, the eluted serum was transferred to the Eppendorf tube and recorded with the name of the patient. The collected sera were stored at -20°C until analysis. Serum with hemolysis or lipemic appearance was not included in the study.

### Measurement of Serum Ischemia Modified Albumin Level

Serum samples were collected and analyzed in the ELISA section of the laboratory. Also, human IMA kits belonging to the company that analyzed the serum samples were used by the kit usage protocols.

### Statistical Data

The IBM SPSS Statistics for Windows version 21.0 software (IBM Corp., Armonk, NY, USA) was used for statistical analysis. The Shapiro-Wilk normality test was performed to determine which tests were appropriate for the available dataset. For the data with normal distribution, parametric tests were performed, and nonparametric tests were performed for the data with no normal distribution. In a comparison of the parametric data one-way analysis of variance (ANOVA) test was used, as well as a posthoc test (Dunnett T3). In a comparison of nonparametric data, the Kruskal Wallis test was used for multiple comparisons, and the Mann-Whitney U test was used for paired comparisons. Averages were given as mean ± standard deviation (SD). *P*-values were statistically significant when calculated below 0.05.

## Results

**Fig. 2:**
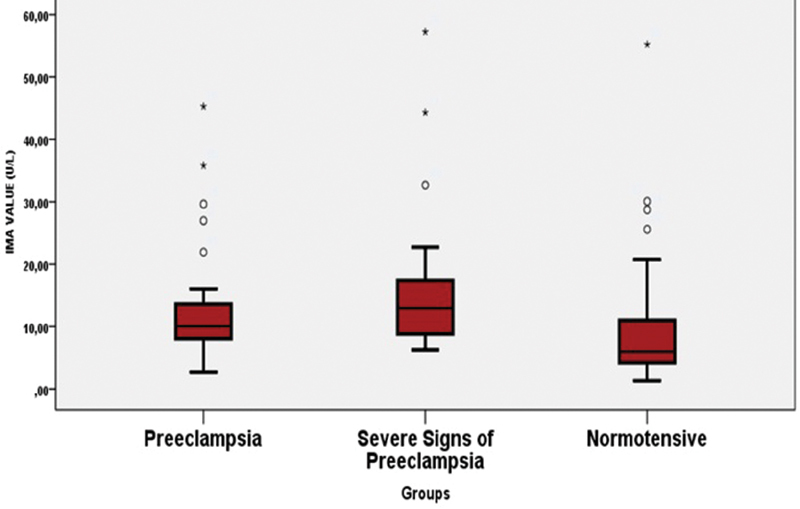
Comparison of groups in terms of ischemia-modified albumin levels.

A total of 90 patients were included in the study and 30 patients were in each group. Group 1 consisted of preeclamptic patients with signs of severity; group 2 was preeclamptic patients; and group 3 comprised normotensive patients. When the demographic characteristics of the patients were examined, a statistically significant difference was found between the groups in terms of age, gestational age, and blood pressure (Table 1). The mean age of the patients in the preeclamptic with signs of severity group was higher than in the normotensive group (30.0 ± 7.1 vs. 26.0 ± 4.3; *p* = 0.033). On the other hand, the mean age was not significantly different between the two preeclampsia groups. Gestational age was lower in the preeclampsia group with signs of severity than the preeclampsia and normotensive groups. (*p* = 0.004). There was no difference between preeclampsia and normotensive groups in terms of gestational age. Systolic and diastolic blood pressures were significantly different in all 3 groups (p < 0.001). Both systolic and diastolic blood pressures were highest in the preeclampsia with the severe features group, followed by preeclampsia and lowest in the normotensive group (p < 0.001) (Table 1). The levels of IMA were determined as follows: 15.7 ± 11.2 U / L in the preeclampsia with severe features group; 13.2 ± 9.5 U / L in the preeclampsia group and 10.2 ± 11.5 U / L in the normotensive group. The IMA levels were significantly lower in the normotensive group compared with the other two groups. In terms of the IMA value, there was a statistically significant difference between the normotensive and the preeclampsia groups (*p* = 0.003). The statistically significant difference was found between the normotensive and the preeclampsia with signs of the severity groups too p < 0.001. There was no statistically significant difference between pregnant women with preeclampsia and preeclampsia with severe features in terms of IMA (*p* = 0.191) (Fig. 2). No statistically significant difference was found between the groups in terms of gravidity, parity, number of miscarriages, number of children living, weight, height and body mass index (BMI) (*p* > 0.05) (Table 1). There was no significant difference between the groups in terms of hemoglobin, white blood cell, platelet, alanine transaminase, aspartate transaminase and urea levels (*p* > 0.05) (Table 2). A significant difference was found between the groups in terms of creatinine levels, and this difference was statistically significant in terms of the severity of preeclampsia (*p* = 0.014).

**Table 1:** Comparison of the groups in terms of demographic and examination findings

Variables	Preeclampsia with signs of severity (*n* = 30)	Preeclampsia (*n* = 30)	Normotensive (*n* = 30)	*P*-value
Age	30.0 ± 7.1^a^	29.2 ± 6.7	26.0 ± 4.3^a^	0.033
Gravidity	2.5 ± 1.8	2.4 ± 1.5	2.3 ± 1.0	0.922
Parity	1.1 ± 1.4	1.1 ± 1.2	0.8 ± 0.9	0.768
Miscarriage	0.4 ± 1.1	0.3 ± 0.6	0.4 ± 0.7	0.284
Number of children living	1.1 ± 1.4	1.1 ± 1.2	0.8 ± 0.9	0.720
Weight (kg)	83.3 ± 12.0	85.9 ± 10.9	80.4 ± 8.5	0.146
Height (cm)	159.8 ± 6.1	159.3 ± 7.9	157.9 ± 8.3	0.620
BMI (kg/m^2^)	32.7 ± 5.6	34.0 ± 5.6	32.5 ± 4.8	0.488
Gestational age	35.2 ± 2.9^b,c^	37.2 ± 2.0^b^	36.8 ± 1.9^c^	0.004
Systolic BP (mmHg)	166.3 ± 12.9^d^	147.3 ± 8.6^d^	115.6 ± 7.7^d^	< 0.001
Diastolic BP (mmHg)	111.6 ± 10.5^e^	93.6 ± 5.5^e^	67.6 ± 8.1^e^	< 0.001

Abbreviations: BMI, body mass index; BP, blood pressure; cm, centimeter; kg, kilogram; m2, square meters; mmHg, millimeters of mercury.

Data are given as mean ± standard deviation (SD); Exponential letters (a, b, c, d, e) which show that the statistically significant differences between groups; p-values for (a, b, c, d, e), respectively; *p* = 0.033, *p* = 0.012, *p* = 0.047, p < 0.001, p < 0.001 was determined.

**Table 2:** Comparison of groups in terms of laboratory findings

Variables	Preeclampsia with signs of severity (*n* = 30)	Preeclampsia (*n* = 30)	Normotensive (*n* = 30)	*P*-value
Hemoglobin (g/dl)	11.4 ± 1.7	11.0 ± 1.5	11.4 ± 1.2	0.630
White blood cell (x10^3^/mm^3^)	12.0 ± 4.5	11.5 ± 4.8	11.9 ± 2.6	0.860
Platelets (x10^3^/mm^3^)	234.3 ± 73.4	242.9 ± 61.5	244.4 ± 84.0	0.849
AST (U/L)	43.3 ± 65.8	24.2 ± 8.3	26.3 ± 10.2	0.634
ALT(U/L)	30.5 ± 50.8	14.7 ± 7.6	11.5 ± 4.3	0.321
Urea (mg/dl)	11.1 ± 6.5	9.2 ± 3.7	8.0 ± 2.5	0.187
Creatinine (mg/dl)	0.8 ± 0.4^a^	0.6 ± 0.1	0.6 ± 0.1^a^	**0.036**

Abbreviations: ALT, alanine aminotransferase; AST, aspartate aminotransferase; G/dl, gram/deciliter; mg/dl, milligram/deciliter; mm3, millimeter cube; U/L, unit/liter.

Data are given as mean ± standard deviation (SD); Exponential letter (a) which shows the statistically significant differences between groups; p-value for (a); *p* = 0.014 was determined.

## Discussion

Preeclampsia is a disorder characterized by extensive vascular endothelial damage and vasospasm that occurs after the 20th week of pregnancy and continues until 4–6 weeks after birth. Clinically, hypertension and proteinuria are defined with or without pathological edema.^9^

Many reasons, such as an abnormal trophoblastic invasion of uterine blood vessels, vascular endothelial damage, placental ischemia, diffuse vasospasm, abnormal nitric oxide and lipid metabolism, oxidative stress, coagulation anomalies, immunological intolerance between maternal and fetoplacental tissue, genetic and nutritional factors, are attributed for etiology of preeclampsia.^10^

A hypoxic intrauterine environment is required for early trophoblast development in normal pregnancies. In this context, maternal serum IMA levels increased, reflecting the oxidative stress associated with placental development. The average serum value of IMA rises significantly during the pregnancy according to what is reported in the literature.^11^ On the other hand, maternal serum IMA levels increased in the first trimester in women with preeclampsia and clinical signs of defective endovascular trophoblast development.^12^

In a prospective study, it has been reported that preeclampsia develops in patients with high IMA levels between 11 to 14 weeks and this is a result of abnormal trophoblastic invasion.^13^ In 2010, Üstün et al^3^ have studied 18 normotensive, 18 preeclamptic and 18 preeclamptic women that includes severe features, and the gestational age, pregnant age, and BMI were not significantly different between the groups. In another study, 19 normotensive and 19 preeclamptic pregnant women were evaluated for serum IMA levels, which were measured by ELISA, as in our study; there was no statistically significant difference in age and pregnant age between these groups.^14^

In our study preeclampsia severity was significantly associated with age. The mean age of the women with severe features of preeclampsia was the highest. In terms of gestational age, preeclamptic women with signs of severity presented the lowest number. There was no difference between the preeclampsia and the normotensive groups in terms of gestational age. Both systolic and diastolic blood pressures were highest in the preeclampsia with severe features group (Table 1).

No statistically significant difference was found between the groups in terms of gravidity, parity, number of children living, weight, height, and BMI.

In 2010, Gafsou et al^15^ conducted a study that compared IMA levels in 22 non-pregnant women, 19 healthy pregnancies, and 20 preeclamptic pregnancies. The level of IMA in preeclamptic pregnancies was found to be significantly higher than in the healthy pregnant women, and this difference was maintained until postpartum. In the present study, it was concluded that increased IMA levels in the first trimester could predict the development of preeclampsia.^15^

In 2008, van Rijn et al^6^ evaluated 12 normotensive and 12 preeclamptic patients for serum IMA levels, but no significant correlation could be detected.

In 2016, in a study by Bahinipati and Mohapatra,^8^ it was emphasized that the level of serum IMA increases in normal pregnancy; therefore, continuous monitoring of serum IMA can give an idea about the progression of pregnancy and any deviation or increase in IMA levels may indicate complications in pregnancy.

In our study, there was a significant correlation between preeclampsia groups and healthy pregnant women in terms of IMA levels. The levels of IMA were lower in normotensive patients than in the preeclampsia groups. However, IMA levels were not correlated in terms of the severity of the disease. When the studies in the literature are examined, increasing IMA levels in normal pregnancy compared with non-pregnant women can be increased in preeclamptic pregnant. Monitoring the IMA level and detecting abnormal increase according to the standards may indicate the complications of pregnancy, especially oxidative damage. This may allow earlier intervention to the complications and reduction of morbidity and mortality.

In addition to IMA, different biochemical and hemogram parameters were evaluated in preeclamptic pregnant women. In the study performed by Delic, blood hemogram and biochemistry parameters were compared in 34 preeclamptic and 35 healthy pregnant women; blood urea nitrogen (BUN) and creatinine were found to be high in preeclamptic pregnant women, whereas the platelet count was low.^16^

In our study, a significant difference was found between the groups in terms of creatinine levels and this difference was statistically significant between the preeclampsia with severe features and normotensive groups (Table 2).

According to our data, IMA levels of preeclamptic women increased significantly compared with the normal control group. The lower concentrations of IMA than preeclampsia in normotensive pregnancies when compared with preeclamptic patients suggest that inflammation and endothelial cell activity in preeclampsia may alter the albumin molecule in the plasma of preeclamptic patients and may increase the levels of IMA. Also, oxidative stress and hypoxic environment predominant preeclampsia may cause ROS increase, which may cause several changes in IMA concentrations.

Observing IMA levels may be helpful in order not to overlook the complications of pregnancy, especially those caused by oxidative damage. This may allow earlier intervention to the complications and reduction of morbidity and mortality. When we look at the literature, it is noted that IMA levels arise during pregnancy. On the other hand, IMA levels increase in women with preeclampsia. There are some studies in the literature show that the IMA level is maintained until postpartum. In the literature, we can argue that the IMA molecule partially represents the differences in placental perfusion when looking at similar changes in IMA levels in a limited number of similar studies. Maternal serum IMA levels increased, reflecting the oxidative stress associated with placental development. Ischemia-modified albumin levels have been shown to increase in some pathologies outside of pregnancy;

1. Cerebrovascular-ischemic stroke, subarachnoid and intracranial hemorrhage
2. Peripheral vascular disease—arterial occlusion, deep vein thrombosis, mesenteric infarct
3. End-stage renal disease
4. Advanced liver cirrhosis
5. Acute infections
6. Malignancies
7. Systemic sclerosis
8. Intrauterine disorders—normal pregnancies, endometriosis, complicated deliveries
9. Prostatic diseases—hyperplasia or cancer^17^ ^18^

## Conclusion

As a result, in patients with preeclampsia, serum IMA levels were higher than in healthy pregnant women. However, no significant correlation was determined between serum IMA levels and preeclampsia severity. One of the reasons for this is the fact that preeclampsia is multifactorial; the other possible reason is the limited number of patients. There were some limitations in our study. We studied IMA levels in pregnant women diagnosed with preeclampsia; therefore, we could not research the levels of IMA in the first trimester. So, in this study, we could not examine the course of IMA in the same pregnant women. We did not estimate the serum albumin level in this study so we could not correlate the IMA/albumin ratio. Also we could not estimate IMA in conjunction with other markers of oxidative stress in cord blood, which are known to be increased in preeclampsia. Increases in serum concentrations of IMA suggest that measurements of these biomarkers may perhaps be useful in monitoring pregnancies for the development of preeclampsia. However, more comprehensive, prospective and long-term studies are needed.
